# VigiNUTRI Brasil: methods of request, data extraction, treatment and consistency analysis of individual data from adolescents monitored by the Food and Nutrition Surveillance System (Sisvan Web)

**DOI:** 10.1590/S2237-96222024v33e20231479.en

**Published:** 2024-10-14

**Authors:** Rafaella Lemos Alves, Natacha Toral, Thiago Luiz Nogueira da Silva, Vivian Siqueira Santos Gonçalves

**Affiliations:** 1 Universidade de Brasília, Programa de Pós-graduação em Nutrição Humana, Brasília, DF, Brazil; 2 Universidade Federal do Rio de Janeiro, Instituto de Estudos em Saúde Coletiva, Rio de Janeiro, RJ, Brazil; 3 Universidade de Brasília, Programa de Pós-graduação em Saúde Coletiva, Brasília, DF, Brazil

**Keywords:** Vigilancia Alimentaria y Nutricional, Atención Primaria en Salud, Base de Datos, Enlace de datos, Sistema de Información en Salud., Food and Nutritional Surveillance, Primary Health Care, Database, Data Binding, Health Information Systems

## Abstract

**Objective:**

To describe the methods for requesting, extracting data, processing and analyzing the consistency of anthropometric and food consumption data of adolescents monitored by Sisvan Web.

**Methods:**

Methodological study with individualized data from Sisvan web between 2008 and 2018. The modules of anthropometry and consumption, made available by the Ministry of Health, had a unique identifier for linkages. Implausible values and individuals outside the age range were excluded. Consistency analyses, with corrections for imputations and descriptive statistics, were performed using Stata 16.0 software.

**Results:**

A database was obtained with 18,812,232 observations of anthropometric data between 2008 and 2018 and 440,534 records of food consumption between 2015 and 2018; after merging the banks, 64,976 observations were obtained.

**Conclusion:**

The combination of anthropometry and food consumption databases made it possible to link individual adolescent data and build a database with information for future analyzes relating to the dietary and nutritional profile of the same individual.

## Study contributions

**Main results:**

The proposal for processing individual data from the Food and Nutrition Surveillance System (Sisvan Web) generated 18 million observations for anthropometric data and 65,000 observations after merging the anthropometry and food consumption databases.

**Implications for services:**

This work will enable replication of the methodology for processing data available in Sisvan Web, allowing for enhanced analyzes by healthcare teams and researchers in the field of public health.

**Perspectives:**

It is expected that these procedures will assist researchers, managers and healthcare professionals in handling and analyzing the information generated by Sisvan Web.

## RESUMEN

**Objetivo:**

Describir los métodos de solicitud, extracción de datos, procesamiento y análisis de consistencia de datos antropométricos y consumo alimentario de adolescentes monitoreados por Sisvan Web.

**Métodos:**

Estudio metodológico con datos individualizados del Web Sisvan de 2008 a 2018. Los módulos de antropometría y consumo, dispuestos por el Ministerio de Salud, contaban con identificador único de vinculación. Se excluyeron valores inverosímiles e individuos fuera del rango de edad. Los análisis de consistencia, con correcciones por imputaciones y estadísticas descriptivas, se realizaron utilizando el software Stata 16.0.

**Resultados:**

Se obtuvo una base con 18.812.232 observaciones de datos antropométricos de 2008 a 2018, y 440.534 registros de consumo alimentario de 2015 a 2018; tras la fusión de los bancos se obtuvieron 64.976 observaciones.

**Conclusión:**

La combinación de datos de antropometría y consumo alimentario permitió vincularlos y construir una base de información para futuros análisis del perfil dietético y nutricional del mismo individuo.

## INTRODUCTION

Food and nutrition surveillance, one of the guidelines of the National Food and Nutrition Policy (*Política Nacional de Alimentação e Nutrição* – PNAN), involves the continuous description and trend forecasting in the food and nutritional status of the Brazilian population and their determining factors.^1^ The Food and Nutrition Surveillance (*Vigilância Alimentar e Nutricional* – VAN) serves as an essential management tool, supporting the planning, implementation and evaluation of health actions aimed at improving the country’s food and nutritional status.^2^

Within the Brazilian National Health System (*Sistema Único de Saúde* – SUS), Primary Health Care (PHC) teams are responsible for carrying out food and nutrition surveillance actions. The Ministry of Health recommends that PHC services should at least perform anthropometric evaluations, measuring weight and height, and monitoring food consumption markers for individuals at all stages of life (children, adolescents, adults, older adults and pregnant women), whose data are consolidated and classified in the Food and Nutrition Surveillance System (*Sistema de Vigilância Alimentar e Nutricional* – Sisvan Web) and made available to the population in an aggregated form through an online platform. ^3,4^ Its use, either alone or associated with other health information systems, allows for the identification of priorities according to the dietary and nutritional profile of the population assisted by PHC.^5^

Since its implementation in 2008, there has been significant progress in Sisvan coverage across different regions of the country, suggesting better monitoring of the population’s nutritional status.^6,7^ However, many publications on this topic use only aggregate data from Sisvan Web public reports,^6-8^ which limits the analytical potential of data.^6-11^ Although various publications emphasize the potential of using anthropometric and food consumption data generated by Sisvan Web, ^9-12^ there is a lack of methodological studies detailing the procedures for requesting, processing and analyzing the consistency of individual data available in the system. Methodological rigor at all stages of data processing is crucial to ensure the generation of high-quality information, supporting both descriptive and analytical research.^13^

In this context, the objective of this study was to describe the methods for data request, extraction, processing and consistency analysis of individual data (anthropometric and food consumption) for adolescents monitored by Sisvan Web between 2008 and 2018.

## METHODS

### Study design

This was a methodological study describing the steps for requesting, processing and analyzing the consistency of individual data from Sisvan Web.

### Setting and participants

Sisvan is a health information system established by Ordinance No. 1,156, of August 31, 1990. Its expansion and improvement occurred through its linkage to the Ministry of Health’s assistance programs and following the publication of PNAN in the 1990s, allowing its coverage to extend nationwide. ^3,4^ The system is periodically updated with anthropometry and food consumption data of users at different stages of life monitored in PHC. ^4^ For this study, we analyzed individual anthropometry and food consumption data of adolescents aged 10 to 19 years monitored by PHC between 2008 and 2018, which had a unique identifier assigned by the Brazilian National Health System Information Technology Department *(Departamento de Informática do SUS* – DataSUS) as a key for necessary linkages. It is worth highlighting that the current dietary intake module came into effect in 2015, making it impossible to cover the entire requested period.

This work is part of a broader study entitled “Food and Nutrition Surveillance of the Population Monitored in Primary Health Care – VIGINUTRI Brazil”, which primarily aims to analyze the trajectory of the nutritional status and dietary profile of individuals monitored by PHC at different stages of life, using data obtained from Sisvan Web.

### Variables

The Ministry of Health was requested to provide the anthropometry module database with weight and height variables, as well as the food consumption module with information on dietary behavior and intake,^14^ in addition to demographic data of the adolescents. The variables, along with their respective response categories, are shown in Box 1. 

The AnthroPlus software^15^ was used to calculate the body mass index (BMI) for each individual and the BMI-for-Age and Height-for-Age Z-scores, taking sex into consideration. As per the World Health Organization definition, Z-scores greater than +5 and less than -5 for BMI-for-age; and greater than +5 and less than -6 for Height-for-age were considered implausible^15^. Measurements outside these reference values were excluded. ^15^ Other data requested are shown in Box 1.

### Data source

**Figure 1:**
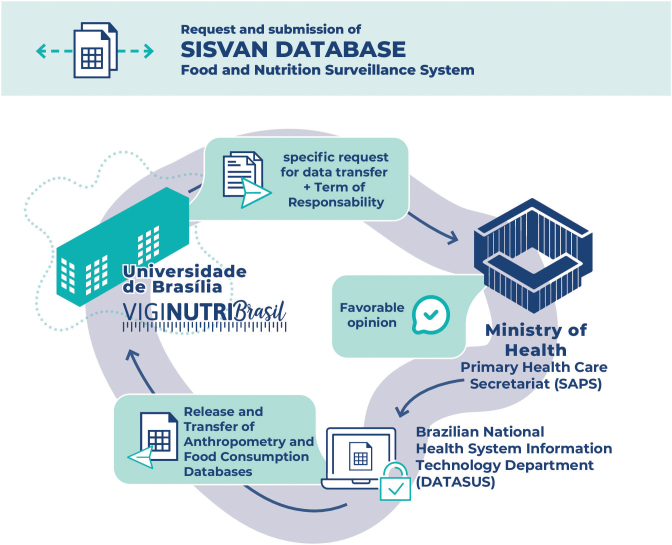
Request flow and acquisition of individual databases from Sisvan Web, 2008-2018

Data were obtained through a specific request to the federal system manager, namely, the Ministry of Health, according to recommendations and provisions of the Access to Information Law (Law No. 12,527, of 11/18/2011) and Ordinance SAS/MS No. 884, of 12/13/2011,^16,17^ which establishes the process for requesting data from national information systems. The data request letter, accompanied by the term of responsibility, was sent to the office of the then Primary Health Care Secretariat, in 2020, which issued a favorable opinion on making the database available. The Brazilian National Health System Information Technology Department *(Departamento de Informática do SUS* – DataSUS) granted access to the database (Figure 1).

Database was sent in “CSV” format. without any nominal identification of users, having only a unique identifier assigned by DataSUS as the key for necessary linkages

### Measures adopted to avoid bias

Implausible anthropometric values and individuals outside the age range were excluded. Furthermore, all variables were checked in order to retrieve information from other observations of the same ID.

### Data access and cleaning techniques

In order to process the data from both modules, anthropometry and food consumption, errors, absences and inconsistencies in the database were investigated. Initially, the database was cleaned, excluding invalid records due to data allocation errors, when the values of one or more variables had been transferred to a single corresponding variable, and records without sex data and with negative age records. For weight and height values, the extremes were manually reviewed.^15^ Manual review, using simple random sampling of around 10 records per block of 100,000 was conducted at each stage comparing with the individual’s set of measurements. Weight and height values were sorted in ascending order in order to check extreme outliers lower than the 1^st^ percentile and higher than the 99^th^ percentile. Weight values less than 10.0 kg and greater than or equal to 150.0 kg, as well as height measurements less than 1.10 m and greater than 2.0 m were investigated step by step, in order to identify an error pattern that would justify exclusion or correction. In these cases, these values were considered implausible for the adolescent cohort, justifying the manual review of the extremes. ^15^ Subsequently, with the exclusion of 2,359,591 outlier values, 674 pairs of duplicate measurements taken on the same date, but with different height values, were identified. As the variability of the difference in more than 95% of the sample was minimal (less than 5 cm), we decided to calculate the average and exclude 337 duplicates.

Z-score for BMI, weight and height adjusted for sex and age was then applied to the valid measurements and the lower and upper standard deviation limits for each indicator were used as signaling limits to identify any extreme or potentially incorrect Z-score values.^15^

Records of individuals with a height measurement error and only one height measurement in the database were disregarded as it was not possible to compare them with others, making it impossible to identify the error pattern.

### Implementation of data linkage between anthropometry and food consumption databases

After linking the data from the two databases, anthropometric and food consumption, the constructed database was considered suitable for performing analyses relating food consumption data to the nutritional status of the population assisted by PHC.

### Statistical methods

Consistency analyses of the database were performed through imputations, descriptive statistics (mean, standard deviation, percentiles, minimum and maximum values), absolute and relative frequencies of sociodemographic and anthropometric variables. All analyses were performed using Stata 16.0 software.

### Ethical considerations

Taking into consideration that the access to the database with individual information on the population monitored by Sisvan Web was necessary and as required by Ordinance SAS/MS No. 884, of 12/13/2011,^17^ the project that originated the study was submitted to and approved by the Research Ethics Committee of the Faculdade de Ciências da Saúde da Universidade de Brasília, under opinion No. 3,798,009 of January 10, 2020; Certificate of Submission for Ethical Appraisal 19024819.3.0000.0030.

## RESULTS 

Three separate datasets were received via internet transfer due to their large volume (21,331,994 anthropometric records and 442,580 food consumption records) and the organizational nature of the system: anthropometry module from 2008-2013, anthropometry module from 2014-2018 and food consumption module from 2015-2018.

### 1. Database preparation: anthropometry module

The Sisvan Web database included 21,331,994 observations from 2008 to 2018, corresponding to data from 11,619,157 individuals.

Due to the large volume of data, and in order to operationalize the analysis, both anthropometry files (2008 to 2012 and 2013 to 2018) were grouped and subdivided into three partitions, according to data availability in each period: 1) data from 2008 to 2012; 2) data from 2013 to 2018; 3) data collected in both periods.

**Box 1:** Variables requested for the anthropometry and food consumption databases

Anthropometry database variables	Food consumption database variables
Weight (kg) Height (cm) Federative Unit and monitoring Municipality with IBGE code Participant’s monitoring region (North; Northeast; Southeast; South; Midwest) Participant identification (key) Neighborhood of residence Date of birth (day/month/year) Sex (female/male) Race/skin color (White; Mixed-race; Black; Indigenous and Asian) Traditional peoples/community of origin of the participant (Quilombola communities; agro-extractivists; Catingueiros; Caiçaras; Fundo e fecho de pasto communities; Cerrado communities; Extractivist commnunities; Faxinalense communities; Geraizeiros; Shellfish gatherers; Pantanal dwellers; Artisanal fishermen; Pomeranians; Roma people; Terreiro communities; Babassu coconut breakers; Riverside dwellers; Rubber tappers, Vazanteiros, Retireiros. Nationality (Brazilian; foreign) Country, Federative Unit and Municipality of birth Attend or have attended school or daycare (yes/no) Highest school year attended or that have attended: Daycare; Preschool; Literacy class; Elementary Education 1st to 4th grades; Elementary Education 5th to 8th grades; Complete Elementary Education; Special Elementary Education; EJA Elementary Education – early years (Supplementary elementary education 1st to 4th); EJA Elementary Education – final years (Supplementary elementary education 5th to 8th); High school; Standard High School (Scientific high school, Technical high school, etc.); Special High School; EJA High School (Supplementary High School Education); Higher Education; Self-improvement course; Specialization course; Master’s degree; Doctorate; Adult Literacy (Mobral, etc.); None; No information provided Linked System/Program (e-SUS/Bolsa Família/Sisvan Web) Name of the healthcare center Place of service (primary healthcare center/mobile health unit/household/school or daycare/institution or shelter/health academy/others) Date of sevice Sickle cell anemia (yes/no) Diabetes mellitus (yes/no) Cardiovascular disease (yes/no) Hypertension (yes/no) Osteoporosis (yes/no) Other diseases (yes/no) Iron deficiency anemia (yes/no) Iodine deficiency (yes/no) Hypovitaminosis A (yes/no)	Federative Unit and Monitoring Municipality with IBGE code Participant’s monitoring region Participant identification (key) Date of birth (day/month/year) and age (days) Sex (female/male) Date of food consumption record Follow-up/care date Linked System/Program (e-SUS/Bolsa Família/ Sisvan Web) Place of service (primary healthcare center/mobile health unit/household/school or daycare/institution or shelter/health academy/others) Habit of eating meals while watching TV, using the computer and/or cell phone (yes/no/don’t know) Which meals do you have throughout the day? Breakfast (yes/no); Morning snack (yes/no); Lunch (yes/no); Afternoon snack (yes/no); Dinner (yes/no); Supper (yes/no); Yesterday, did you consume: Beans (yes/no); Fresh fruit (yes/no); Greens/vegetables (yes/no); Hamburger and/or processed meats (yes/no); Sweetened beverages (yes/no); Instant noodles, packaged snacks or savory biscuits (yes/no); Stuffed biscuit, sweets or treats (yes/no).

Legend: VigiNUTRI Brazil: Food and Nutrition Surveillance of the Population monitored in Primary Health Care; Sisvan Web (Sistema de Vigilância Alimentar e Nutricional): Food and Nutrition Surveillance System.

**Figure 2:**
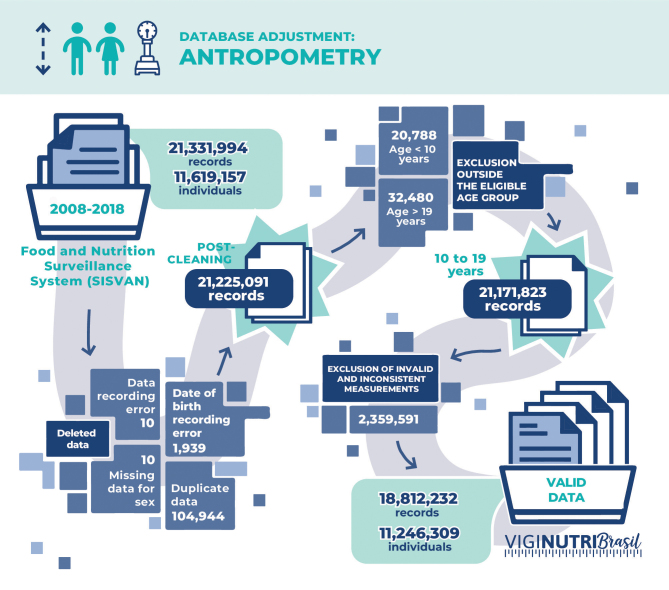
Technical analysis flow and adjustment of the anthropometry database, Sisvan Web, 2008-2018

Then, procedures were adopted to assess quality regarding missing data, inconsistencies and detection of implausible extreme values (Figure 2). At this stage, it was verified whether the nature of the data was valid according to the respective variable. Ten observations with data recording errors were found and excluded.

Subsequently, the existence of 23 observations with missing values for the sex variable was verified, of which 13 were retrieved from other data associated with the same individual identification key and 10 were excluded.

After this initial analysis, 104,944 (0.49%) duplicate observations were excluded, and age was calculated based on the difference between the measurement registration date and the date of birth. 1,939 (0.009%) observations with negative age due to errors in the date of birth or anthropometry registration date were excluded. Then, 53,268 participants outside the study age range were excluded, and the process of standardizing weight and height measurements began.

For that, only seven observations with recording errors in the anthropometric data were identified and special characters in three weight measurements and four height measurements were deleted in order to ensure the numerical format of the anthropometric measurements.

After this process, anthropometric measurements considered implausible were analyzed for inconsistencies.

**Figure 3:**
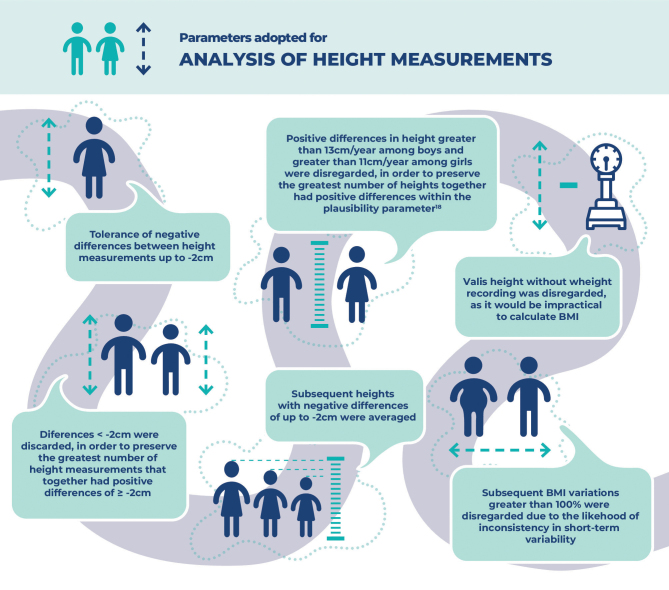
Parameters adopted by the authors for internal consistency analysis between height measurements of the same individual monitored by Sisvan Web, 2008-2018

Finally, an internal consistency analysis was performed between the measurements of the same individual, i.e., consistency in the difference between the heights of the same individual (with two or more measurements) was observed, in order to preserve the greatest number of biologically plausible measures over time.^18^ Based on this premise, the parameters described in Figure 3 were established.

After completing all these steps, 18,812,232 valid measurements were considered for the anthropometry database, of which 4,176,666 (86.38%) were from 2008 to 2018, 6,838,466 (85.61%) between 2008 and 2012 and 7,797,100 (93.42%) between 2013 and 2018.

### 2. Database preparation: food consumption module

**Figure 4:**
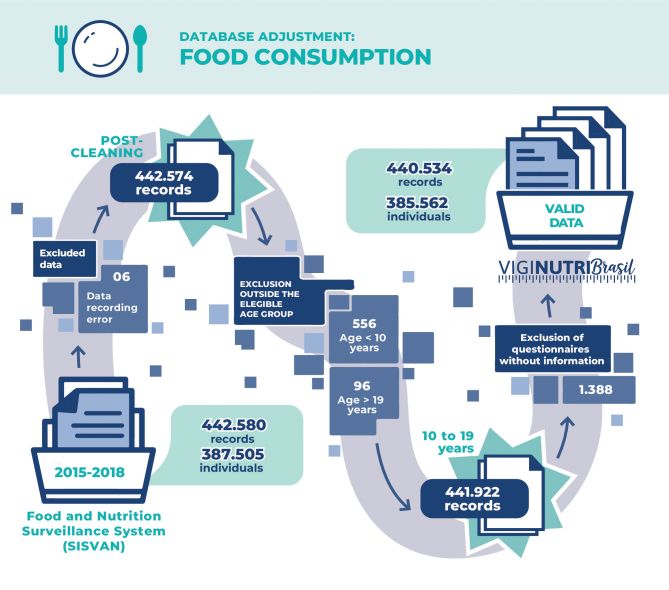
Flow of technical analysis, cleaning and adjustment of the food consumption database, Sisvan Web, 2015-2018

The Sisvan Web database with food consumption data provided by the Ministry of Health covered 442,580 observations from 387,505 individuals between 2015 and 2018. As with the anthropometry database, procedures were adopted to perform technical data analysis (Figure 4). Data with errors and those from individuals outside the required age range, as well as questionnaires without information were excluded (0,31%). Thus, 440,534 records were obtained, of which 182,105 (41.21%) had complete questionnaires and 258,429 (58.48%) had incomplete questionnaires.

### 3. Implementation of data linkage

After the consistency analysis of data, linkage between the anthropometry database (2015-2018), which contained 6,764,155 observations and food consumption database which 440,534 observations, was performed. A total of 385,562 individuals with 654,962 observations of nutritional status and food consumption data were found, based on the individual identification key. However, only 214,428 anthropometric assessments occurred from 2015 to 2018 among those with any food consumption data. 

Merging different databases into one does not necessarily require the individual to present anthropometric and intake data in both databases. For example, despite having the same identification, an individual may only present information about their date of birth or the place of service in one of the databases. Consequently, 242,483 observations were excluded, relating to cases where the individual had no nutritional status data or food consumption data, resulting in a database with 412,479 observations.

The subsequent step was the technical analysis of the food consumption questionnaire in order to select only one questionnaire when an individual had a duplicate questionnaire. After removing duplicate questionnaires (n = 329), 412,150 consumption data were found, and thus, pairs of food consumption questionnaires and anthropometric assessments were formed. Given that these assessments do not always occur on the same date in PHC routine, the difference of up to 30 days between both assessments was taken into account, resulting in 64,976 food consumption and anthropometry questionnaires of 63,186 individuals from 2015 to 2018. As these are data from an11-year cohort, they are based on the number of observations, as there are adolescents with more than one measurement.

## DISCUSSION

In this study, data processing resulted in a database with 18,812,232 observations solely of anthropometric data between 2008 and 2018, in addition to 64,976 observations of adolescents after merging the anthropometry and food consumption databases. These data are highly relevant, as they represent individual information from a cohort of adolescents monitored in PHC over 11 years for anthropometry, and four years for food consumption and anthropometry of the same individual; These data have been little explored by the scientific community thus far.

The data generated by Sisvan Web is a valuable source of information for VAN, which has been incorporated into PHC care routines for monitoring the nutritional status of users.^4,5,19^ Since 2008, the system has advanced in both coverage and data quality, contributing to the development of scientific evidence.^19^ However, the use of data in its entirety remains a challenge for some healthcare professionals and managers, as it requires basic knowledge for analysis and interpretation.

As observed in this study, each stage of data processing reaches a high level of complexity that requires professional qualifications, which may be a factor that hinders the popularization of analyses. Underutilization of data affects the implementation of effective actions, mainly at the municipal level, enabling the prevention of diseases and health conditions related to diet and nutrition.^19^

It is common to use data from Sisvan Web public reports to assess the nutritional status and food consumption of individuals at different stages of life.^4,20^ However, the use of individual data to understand the dietary and nutritional profile of users monitored in PHC and the interaction of their personal and environmental aspects is still incipient.^21^ The ability to link different data sources (e.g., primary care and specialized care data) is crucial to improve health information exchange, decision-making, policy development, as well as product and service development.^22^ This database relationship, also known as linkage, has not been widely disseminated in health surveillance environments yet; and, when used, it often involves secondary data from information systems, but still offers advantages for health services.^13^

In this study, the deterministic linkage used between the anthropometry and food consumption databases involved the presence of common unifying keys in different databases in order to construct a single database with anthropometric and nutritional history.^23^

This strategy of technical analysis and consistency between multiple measurements in cohort databases is essential for ensuring quality, especially when operationalizing large datasets.^13^ It also allows the identification of errors and inconsistencies in data entry, a common limitation among studies involving these types of data.^24^.^25^

For this study, the first database (anthropometry) was used as a reference, and the second (food consumption) as a source of new information. Thus, identifying unique keys between the databases was crucial for tracking information related to the same individual. Matching information allows for specific analyzes to evaluate associations, contributing to a broader perspective of the information, avoiding its isolated use. Based on the variables available for each person in the generated database, it becomes possible to include important covariates that were not present in the original database. These can be obtained from different areas, such as social, environmental and health.^25^

A retrospective cohort study assessed the influence of perinatal factors on the development of obesity in children and adolescents in Southern Brazil using anthropometry data recorded on Sisvan Web and perinatal data recorded on the Live Birth Information System (*Sistema de Informação de Nascidos Vivos* – SINASC). The linkage between the two systems enabled the identification of these risk factors for the development of childhood obesity.^5^

Another study, conducted with individual data of adolescents aged 10 to 19 years taking part in the VIGINUTRI Brazil study in 2018, investigated the association of contextual socioeconomic factors in the municipality of residence, behaviors and food consumption with the prevalence of obesity. In this study, contextual factors of the municipalities were extracted from websites and aggregated into the Sisvan database, which improved the scope of possible analyses. Thus, a higher obesity prevalence was observed in adolescents living in municipalities with higher *per capita* income and who consumed hamburgers and processed meats (markers of ultra-processed food consumption) on the day before the record.^21^

Although the distinct methodologies for linkage in the aforementioned studies^5,21^ are different, it is possible to understand the need for investment and encouragement for the use of public information systems databases, as, in addition to the low cost, they provide a wide range of information from birth to aging, which can be used for territorial assessment, decision making, planning and development of actions. It is worth highlighting that making more efforts and raising awareness are needed to enter data into the system, expanding its total coverage to all stages of life.^24^

Furthermore, investment in technology is essential to ensure adequate computer equipment and internet access, especially in smaller municipalities. Regarding the Ministry of Health, it is essential to maintain and improve the system, avoiding its instability, as the quality of the original databases will determine the quality of the linked databases.^13^

Investment in training and capacity-building for PHC teams should be provided in order to sensitize them to the importance of periodic system registration, not only for the anthropometry module, but also for the food consumption module, which still has a much lower volume of records when compared to anthropometry.

Among the limitations identified, the lack of previous studies stood out, leading researchers to adopt the parameter of a 30-day difference between records of monitored adolescents for technical analysis and database adjustments. It is suggested that this be tested in the future. Another limitation is the infeasibility of inspecting all records due to the large number and loss of a lot of data due to low coverage, incorrect data entry and discontinuity in data submission. Although there are limitations, this study stands out for being the first to present a description of all stages of processing individual data from Sisvan, and it is expected that this will be the starting point for the analysis of individual data at other stages of life.

By merging the anthropometry and the food consumption databases of adolescents monitored by Sisvan Web between 2008 and 2018, it was possible to link and aggregate individual data, generating a database with information on dietary and nutritional profile for the same individual.

Sisvan is recognized as a fundamental tool for managing PNAN in the country. The use of its information for action is essential to expand population coverage, focusing on providing subsidies for crucial health policies that support decision-making by professionals and managers. The description provided in this study enables research activities, descriptive and analytical studies that can offer an effective overview of the epidemiological profile of the population monitored in PHC.
