# Supplementary material for: VigiNUTRI Brasil: methods of request, data extraction, treatment and consistency analysis of individual data from adolescents monitored by the Food and Nutrition Surveillance System (Sisvan Web)
Source: Epidemiol Serv Saude. 2024 Oct 14;33:e20231479. doi: 10.1590/S2237-96222024v33e20231479.en (PMC11473065; doi:10.1590/S2237-96222024v33e20231479.en)
Supplement: Translation

# Translation: VigiNUTRI Brasil: métodos de solicitação, extração de dados, tratamento e análise de consistência de dados individualizados de adolescentes acompanhados pelo Sistema de Vigilância Alimentar e Nutricional (Sisvan Web)

**DOI:** 10.1590/S2237-96222024v33e20231479.pt

**Authors:** Rafaella Lemos Alves, Natacha Toral, Thiago Luiz Nogueira da Silva, Vivian Siqueira Santos Gonçalves

**Affiliations:** 1 Universidade de Brasília, Programa de Pós-graduação em Nutrição Humana, Brasília, DF, Brasil; 2 Universidade Federal do Rio de Janeiro, Instituto de Estudos em Saúde Coletiva, Rio de Janeiro,; 3 Universidade de Brasília, Programa de Pós-graduação em Saúde Coletiva, Brasília, DF, Brasil

**Keywords:** Vigilância Alimentar e Nutricional, Atenção Primária à Saúde, Base de Dados, Vinculação de Dados, Sistema de Informação em Saúde

## Abstract

**Objetivo:**

Descrever os métodos para a solicitação, extração de dados, tratamento e análise de consistência de dados antropométricos e de consumo alimentar de adolescentes acompanhados pelo Sisvan Web.

**Métodos:**

Estudo metodológico com dados individualizados do Sisvan Web entre 2008 e 2018. Os módulos de antropometria e consumo, disponibilizados pelo Ministério da Saúde, possuíam um identificador único para *linkages*. Excluíram-se valores implausíveis e indivíduos fora da faixa etária. Análises de consistência, com correções por imputações e estatísticas descritivas, foram realizadas pelo *software* Stata 16.0.

**Resultados:**

Obteve-se uma base com 18.812.232 observações de dados antropométricos entre 2008 e 2018 e 440.534 registros de consumo alimentar entre 2015 e 2018; após a junção dos bancos, foram obtidas 64.976 observações.

**Conclusão:**

A junção dos bancos de antropometria e consumo alimentar permitiu vincular dados individuais dos adolescentes e construir um banco com informações para análises futuras relativas ao perfil alimentar e nutricional do mesmo indivíduo.

## Contribuições do estudo

**Principais resultados:**

A proposta para o tratamento dos dados individualizados do Sistema de Vigilância Alimentar e Nutricional (Sisvan Web) gerou 18 milhões de observações de dados antropométricos e 65 mil observações após a junção dos bancos de antropometria e consumo alimentar.

**Implicações para os serviços:**

Este trabalho permitirá a replicação da metodologia para tratamento dos dados disponíveis no Sisvan Web, possibilitando o aprimoramento das análises pelas equipes de saúde e por pesquisadores do campo da saúde coletiva.

**Perspectivas:**

Espera-se que os procedimentos possam auxiliar pesquisadores, gestores e profissionais da área da saúde com a manipulação e análise das informações geradas pelo Sisvan Web.

## INTRODUÇÃO

A vigilância alimentar e nutricional, uma das diretrizes da Política Nacional de Alimentação e Nutrição (PNAN), consiste na descrição contínua e na predição de tendências das condições de alimentação e nutrição da população brasileira e seus fatores determinantes.¹ Configura-se como uma ferramenta essencial de gestão, subsidiando o planejamento, a execução e a avaliação de ações em saúde focadas na melhoria da situação alimentar e nutricional do país.^2^

No âmbito do Sistema Único de Saúde (SUS), cabe às equipes da Atenção Primária à Saúde (APS) a realização das ações de vigilância alimentar e nutricional. O Ministério da Saúde orienta que, nos serviços de APS, sejam realizadas, no mínimo, a avaliação antropométrica, com a aferição de peso e estatura, e de marcadores do consumo alimentar de indivíduos de todas as fases do curso da vida (crianças, adolescentes, adultos, idosos e gestantes), cujos dados são consolidados e classificados no Sistema de Vigilância Alimentar e Nutricional (Sisvan Web) e disponibilizados à população de forma agregada, por meio de sítio eletrônico.^3,4^ A sua utilização de forma isolada, ou associada a outros sistemas de informação em saúde, permite a identificação de prioridades de acordo com o perfil alimentar e nutricional da população assistida pela APS.^5^

Desde sua implementação, em 2008, houve um grande avanço da cobertura do Sisvan nas diferentes regiões do país, o que sugere um melhor monitoramento da situação nutricional da população.^6,7^ Contudo, muitas das publicações que abordam essa temática utilizam apenas os dados agregados, ou seja, provenientes dos relatórios públicos do Sisvan Web,^6-8^ o que limita o potencial analítico dos dados.^6-11^ Embora diferentes publicações reforcem a potencialidade do uso de dados antropométricos e de consumo alimentar gerados pelo Sisvan Web,^9-12^ há escassez de estudos metodológicos que apresentem os procedimentos necessários para a solicitação, tratamento e análise de consistência dos dados individualizados disponíveis no sistema. O rigor metodológico em todas as etapas de tratamento de dados é de suma importância para garantir a geração de informações de qualidade, subsidiando a realização de investigações descritivas e analíticas.^13^

Nesse sentido, objetivou-se descrever os métodos para a solicitação, extração de dados, tratamento e análise de consistência de dados individualizados (antropométricos e de consumo alimentar) de adolescentes acompanhados pelo Sisvan Web entre 2008 e 2018.

## MÉTODOS

### Desenho do estudo

Trata-se de estudo metodológico de descrição das etapas para solicitação, tratamento e análise de consistência de dados individualizados do Sisvan Web. 

### Contexto e participantes

O Sisvan é um sistema de informação em saúde instituído pela Portaria nº 1.156, de 31 de agosto de 1990. Sua ampliação e aperfeiçoamento se deram a partir da vinculação a programas assistenciais do Ministério da Saúde e após a publicação da PNAN na década de 1990, permitindo que sua cobertura fosse estendida para todo o país.^3,4^ O sistema é alimentado periodicamente com dados de antropometria e consumo alimentar de usuários nas diferentes fases da vida acompanhados na APS.^4^ Para este estudo, foram analisados dados individualizados de antropometria e consumo alimentar de adolescentes de 10 a 19 anos acompanhados pela APS entre os anos de 2008 e 2018, os quais possuíam o identificador único atribuído pelo DataSUS como chave para a realização dos *linkages* necessários. Ressalta-se que o módulo atual de consumo iniciou a sua vigência em 2015, impossibilitando a abrangência de todo o período solicitado. 

Este trabalho integra estudo mais amplo, intitulado “Vigilância Alimentar e Nutricional da População acompanhada na Atenção Primária à Saúde – Viginutri Brasil”, que tem como objetivo principal analisar a trajetória do estado nutricional e do perfil alimentar de indivíduos acompanhados pela APS em diferentes fases do curso da vida, por meio dos dados obtidos pelo Sisvan Web. 

### Variáveis 

Solicitou-se ao Ministério da Saúde o banco de dados do módulo de antropometria com variáveis de peso e altura, e também o módulo de consumo alimentar com informações sobre comportamento e consumo alimentar,^14^ além de dados demográficos dos adolescentes. As variáveis, com suas respectivas categorias de respostas, constam no Quadro 1. 

**Quadro 1:** Variáveis solicitadas para os bancos de dados de antropometria e de consumo alimentar

Variáveis do banco de antropometria	Variáveis do banco de consumo alimentar
Peso (kg) Altura (cm) Unidade da Federação e município de acompanhamento com código do IBGE Região de acompanhamento do participante (Norte; Nordeste; Sudeste; Sul; Centro-Oeste) Identificação do participante (em chave) Bairro de residência Data de nascimento (dia/mês/ano) Sexo (feminino/masculino) Raça/cor da pele (branca; parda; preta; indígena e amarela) Povo/comunidade tradicional de origem do participante (povos quilombolas; agroextrativistas; catingueiros; caiçaras; comunidades de fundo e fecho de pasto; comunidades do Cerrado; extrativistas; faxinalenses; geraizeiros; marisqueiros; pantaneiros; pescadores artesanais; pomeranos; povos ciganos; povos de terreiro; quebradeiras de coco de babaçu; retireiros; ribeirinhos; seringueiros; vazanteiros) Nacionalidade (brasileira; estrangeira) País, Unidade de federação e município de nascimento Frequenta ou frequentou escola ou creche (sim/não) Ano escolar mais elevado que frequenta ou frequentou: Creche; Pré-escola; Classe de alfabetização; Ensino fundamental 1ª a 4ª séries; Ensino fundamental 5ª a 8ª séries; Ensino Fundamental Completo; Ensino fundamental especial; Ensino fundamental EJA - séries iniciais (Supletivo 1ª a 4ª); Ensino fundamental EJA - séries finais (Supletivo 5ª a 8ª); Ensino médio; Médio 2º ciclo (Científico, Técnico etc.); Ensino médio especial; Ensino médio EJA (Supletivo); Superior, Aperfeiçoamento, Especialização, Mestrado, Doutorado; Alfabetização para adultos (Mobral etc.); Nenhum; Sem informação Sistema/Programa vinculado (e-SUS/Bolsa Família/Sisvan Web) Nome do estabelecimento de saúde Local de atendimento (unidade básica de saúde/unidade móvel/domicílio/escola ou creche/instituição ou abrigo/academia de saúde/outros) Data de atendimento Anemia falciforme (sim/não) Diabetes mellitus (sim/não) Doença cardiovascular (sim/não) Hipertensão (sim/não) Osteoporose (sim/não) Outras doenças (sim/não) Anemia ferropriva (sim/não) Deficiência de iodo (sim/não) Hipovitaminose A (sim/não)	Unidade da Federação e município de acompanhamento com código do IBGE Região de acompanhamento do participante Identificação do participante (em chave) Data de nascimento (dia/mês/ano) e Idade (dias) Sexo (feminino/masculino) Data de registro da ficha de consumo alimentar Data de acompanhamento/atendimento Sistema/Programa vinculado (e-SUS/Bolsa Família/Sisvan Web) Local de atendimento (unidade básica de saúde/unidade móvel/domicílio/escola ou creche/instituição ou abrigo/academia de saúde/outros) Costume de realizar refeições assistindo à TV, mexendo no computador e/ou celular (sim/não/não sabe) Quais refeições você faz ao longo do dia? Café da manhã (sim/não); Lanche da manhã (sim/não); Almoço (sim/não); Lanche da tarde (sim/não); Jantar (sim/não); Ceia (sim/não); Ontem, você consumiu: Feijão (sim/não); Frutas frescas (sim/não); Verduras/legumes (sim/não); Hambúrguer e/ou embutidos (sim/não); Bebidas adoçadas (sim/não); Macarrão instantâneo, salgadinhos de pacote ou biscoitos salgados (sim/não); Biscoito recheado, doces ou guloseimas (sim/não).

Legenda: VigiNutri Brasil: Vigilância Alimentar e Nutricional da População acompanhada na Atenção Primária à Saúde; Sisvan Web: Sistema de Vigilância Alimentar e Nutricional.

Por meio do *software* AnthroPlus,^15^ foram calculados o índice de massa corporal (IMC) de cada indivíduo e o escore-Z do IMC para idade e estatura para idade, considerando o sexo. Conforme definição da Organização Mundial da Saúde, foram considerados implausíveis escores-Z superiores a +5 e inferiores a -5 para o IMC para a idade; e superiores a +5 e inferiores a -6 para estatura para a idade^15^ A partir desses limites, foram excluídas as medidas fora desses valores de referência.^15^ Os demais dados solicitados constam no Quadro 1. 

### Fonte de dados

Os dados foram obtidos por meio de solicitação específica com o gestor federal do sistema, a saber, o Ministério da Saúde, segundo o recomendado e disposto na Lei de Acesso à Informação (Lei nº 12.527, de 18/11/2011) e na Portaria SAS/MS nº 884, de 13/12/2011,^16,17^ a qual estabelece o fluxo para a solicitação de cessão de dados dos bancos nacionais dos sistemas de informação. A carta de solicitação para a cessão de dados, acompanhada do termo de responsabilidade, foi enviada ao gabinete da então Secretaria de Atenção Primária à Saúde, no ano de 2020, que emitiu parecer favorável à disponibilização do banco de dados. Em seguida, o Departamento de Informática do Sistema Único de Saúde (DataSUS) procedeu à liberação do acesso ao banco de dados (Figura 1).

**Figura 1:** Fluxo de solicitação e obtenção das bases de dados individualizados do Sisvan Web, 2008-2018

A base foi enviada no formato “csv.”, sem qualquer identificação nominal dos usuários, tendo somente um identificador único atribuído pelo DataSUS como chave para a realização de *linkages* necessários. 

### Medidas adotadas para evitar vieses

Foi realizada a exclusão de valores antropométricos implausíveis, e também de indivíduos fora da faixa etária. Além disso, todas as variáveis foram checadas, com o objetivo de recuperar informações a partir de outras observações de um mesmo ID. 

### Acesso aos dados e métodos de limpeza

Para o tratamento dos dados de ambos os módulos, antropometria e consumo alimentar, foi prevista a investigação de erros, ausências e inconsistências nas informações do banco. Inicialmente foi realizada a limpeza do banco, excluindo-se os registros inválidos por erro de alocação do dado, quando os valores de uma ou mais variáveis haviam sido transportados para uma única variável correspondente, e os registros sem dados sobre sexo e com registro de idade negativa. Para os valores de peso e altura, foi realizada a revisão manual dos extremos.^15^ A revisão manual, por amostragem aleatória simples, de cerca de 10 registros a cada bloco de 100 mil, foi realizada em cada etapa, comparando-se com o conjunto de medidas do indivíduo. Ordenaram-se os valores de peso e altura em ordem crescente, a fim de se checarem os valores extremos discrepantes menores que o percentil 1 e maiores que o percentil 99. Valores de peso menores que 10,0 kg e maiores ou iguais a 150,0 kg, além das medidas de altura menores que 1,10 m e maiores que 2,0 m, foram investigadas etapa por etapa, a fim de se identificar um padrão de erro que justificasse a exclusão ou correção. Nesses casos, esses valores foram considerados implausíveis para a coorte de adolescentes, justificando a revisão manual dos extremos.^15^ Posteriormente, com a exclusão dos 2.359.591 valores discrepantes, foram identificados 674 pares de duplicatas de medidas realizadas na mesma data, mas com valores de altura diferentes. Como a variabilidade da diferença em mais de 95% da amostra foi mínima (menos de 5 cm), optou-se por calcular a média e excluir 337 duplicatas.

Aplicou-se, então, o escore-Z de IMC, de peso e de altura ajustados por sexo e idade nas medidas válidas, e foram usados os limites-padrão inferior e superior do desvio-padrão de cada indicador como limites de sinalização, para se identificarem quaisquer valores Z-score extremos ou potencialmente incorretos.^15^

Registros de indivíduos com erro na medida de estatura e com uma única medida de estatura no banco foram desconsiderados, por não possibilitarem a comparação com as demais, não sendo possível identificar o padrão de erro.

### Implementação da vinculação entre os bancos de antropometria e consumo alimentar

Após a vinculação dos dados dos dois bancos, de informações antropométricas e de consumo alimentar, o banco construído foi considerado apto para a realização de análises, relacionando-se os dados de consumo alimentar com os de estado nutricional da população assistida pela APS.

### Métodos estatísticos 

Foram realizadas análises de consistência do banco, por meio de correções por imputações, estatísticas descritivas (média, desvio-padrão, percentis, valores mínimos e máximos), frequência absoluta e relativa das variáveis sociodemográficas e antropométricas. Todas as análises foram realizadas utilizando-se o *software* Stata 16.0.

### Considerações éticas

Considerando que era necessário o acesso ao banco de dados com informações individualizadas da população acompanhada pelo Sisvan Web e por se tratar de uma exigência descrita na Portaria SAS/MS nº 884, de 13/12/2011,^17^ o projeto que deu origem ao estudo foi submetido e aprovado pelo Comitê de Ética em Pesquisas com Seres Humanos da Faculdade de Ciências da Saúde da Universidade de Brasília, por meio do parecer nº 3.798.009, de 10 de janeiro de 2020; Certificado de Apresentação de Apreciação Ética 19024819.3.0000.0030.

## RESULTADOS 

Foram recebidos, por meio de transferência via internet, três conjuntos de dados distintos, devido ao seu grande volume (21.331.994 registros de antropometria e 442.580 registros de consumo alimentar) e à natureza organizacional do sistema: módulo de antropometria de 2008-2013, módulo de antropometria de 2014-2018 e módulo de consumo alimentar de 2015-2018. 

### 1. Preparação do banco de dados: módulo de antropometria

O banco de dados do Sisvan Web abrangeu 21.331.994 observações no período de 2008 a 2018, que corresponderam aos dados de 11.619.157 indivíduos. 

Devido ao grande volume de dados, e com o intuito de operacionalizar a análise, os dois arquivos de antropometria (2008 a 2012 e 2013 a 2018) foram agrupados e subdivididos em três partições, de acordo com a disponibilidade de dados em cada período: 1) dados de 2008 a 2012; 2) dados de 2013 a 2018; 3) dados coletados em ambos os períodos.

Em seguida, foram adotados os procedimentos para avaliar a qualidade em relação aos dados ausentes, inconsistências e detecção de valores extremos implausíveis (Figura 2). Nessa etapa foi verificado se a natureza do dado seria válida de acordo com a respectiva variável. Foram encontradas 10 observações com erro de registro de dados, que foram excluídas. 

**Figura 2:** Fluxo de análise técnica e ajuste do banco de dados de antropometria, Sisvan Web, 2008-2018

Posteriormente, foi verificada a existência de 23 observações com valores faltantes para a variável sexo, das quais 13 foram recuperadas a partir dos demais dados associados à mesma chave de identificação do indivíduo e 10 excluídas. 

Após essa análise inicial, foram excluídas 104.944 (0,49%) observações duplicadas e calculada a idade a partir da diferença entre a data do registro das medidas e a data de nascimento. Foram excluídas 1.939 (0,009%) observações com idade negativa, por erro na data de nascimento ou na data de registro da antropometria. Em seguida, os 53.268 participantes com idade fora da faixa etária do estudo foram excluídos, e então iniciou-se o processo de padronização das medidas de peso e altura.

Para isso, foram identificadas apenas sete observações com erro de registro nos dados antropométricos e deletaram-se os caracteres especiais em três medidas de peso e em quatro medidas de altura, de forma a assegurar o formato numérico das medidas antropométricas. 

Após esse processo, foi realizada a análise de inconsistências das medidas antropométricas consideradas implausíveis. 

Por fim, foi realizada uma análise de consistência interna entre as medidas do mesmo indivíduo, ou seja, foi observada consistência da diferença entre as alturas do mesmo indivíduo (com duas ou mais medidas), a fim de se preservar o maior número de medidas com plausibilidade biológica ao longo do tempo.^18^ A partir dessa premissa, foram estabelecidos os parâmetros descritos na Figura 3. 

**Figura 3:** Parâmetros adotados pelos autores para a análise de consistência interna entre as medidas de altura do mesmo indivíduo acompanhado pelo Sisvan Web, 2008-2018

Ao finalizar todas essas etapas, foram consideradas 18.812.232 medidas válidas para o banco de antropometria, sendo 4.176.666 (86,38%) do período de 2008 a 2018, 6.838.466 (85,61%) entre 2008 e 2012 e 7.797.100 (93,42%) entre 2013 e 2018. 

### 2. Preparação do banco de dados: módulo de consumo alimentar

O banco de dados do Sisvan Web com os dados de consumo alimentar apresentado pelo Ministério da Saúde abrangia 442.580 observações de 387.505 indivíduos no período de 2015 a 2018. Assim como no banco de dados de antropometria, foram adotados procedimentos para realizar a análise técnica de dados (Figura 4). Foram excluídos dados com erro e aqueles provenientes de indivíduos com idade fora da faixa etária solicitada, bem como questionários sem informações (0,31%). Dessa forma, foram obtidos ao final 440.534 registros, dos quais 182.105 (41,21%) apresentaram o questionário completo e 258.429 (58,48%) possuíam questionários incompletos. 

**Figura 4:** Fluxo de análise técnica, limpeza e ajuste do banco de dados de consumo alimentar, Sisvan Web, 2015-2018

### 3. Implementação da vinculação

Após a análise de consistência dos dados, foi realizada a vinculação entre os bancos de antropometria (2015-2018), o qual continha 6.764.155 observações, e consumo alimentar, com 440.534 observações. Foram encontrados 385.562 indivíduos, com 654.962 observações de dados do estado nutricional e consumo alimentar, a partir da chave de identificação do indivíduo. Contudo, apenas 214.428 avaliações antropométricas ocorreram no período de 2015 a 2018 entre aqueles com algum dado de consumo alimentar. 

O método de anexar as diferentes bases em uma única não necessariamente exige que o indivíduo apresente dados de antropometria e consumo em ambas as bases. Por exemplo, apesar de apresentar a mesma identificação, o indivíduo pode apresentar apenas a informação de sua data de nascimento ou o local de atendimento em uma das bases. Por conta disso, foi necessário excluir 242.483 observações, relativas a casos em que o indivíduo não apresentava nenhum dado de estado nutricional ou nenhum dado de consumo alimentar, resultando em um banco de dados com 412.479 observações.

A etapa posterior foi a análise técnica do questionário de consumo alimentar, de modo a selecionar apenas um único questionário quando algum indivíduo apresentasse um questionário duplicado. Após a limpeza de questionários duplicados (n = 329), foram encontrados 412.150 dados de consumo, e assim formaram-se os pares de questionário de consumo e a avaliação antropométrica. Uma vez que, na rotina da APS, essas avaliações constantemente não acontecem na mesma data, levou-se em consideração a diferença de até 30 dias entre a realização de ambos, resultando em 64.976 questionários de consumo alimentar e antropometria de 63.186 indivíduos, no período de 2015 a 2018. Por se tratar de dados de uma coorte de 11 anos, esses são baseados no número de observações, pois há adolescentes que apresentam mais de uma medida. 

## DISCUSSÃO

Neste estudo, o tratamento dos dados resultou em uma base com 18.812.232 observações apenas de dados antropométricos entre 2008 e 2018, além de 64.976 observações de adolescentes após a junção dos bancos de antropometria e consumo alimentar. Esses dados são de grande relevância, uma vez que representam informações individualizadas de uma coorte de adolescentes, acompanhados na APS ao longo de 11 anos para a parte de antropometria, e quatro anos para o consumo alimentar e antropometria de um mesmo indivíduo; dados esses, até então, pouco explorados pelo meio científico. 

Os dados gerados pelo Sisvan Web constituem uma boa fonte de informações para a VAN, que foi incorporada às rotinas de atendimento na APS para o monitoramento do estado nutricional dos usuários.^4,5,19^ A partir de 2008, o sistema avançou tanto na cobertura quanto na qualidade dos dados, contribuindo para a construção de evidências científicas.^19^ No entanto, ainda é um desafio para uma parte de profissionais de saúde e gestores a utilização dos dados em sua completude, uma vez que requerem conhecimento básico para a análise e interpretação. 

Conforme observado neste estudo, o tratamento de dados em cada etapa alcança um alto nível de complexidade que exige qualificação profissional, podendo ser um fator dificultador para a popularização das análises. A subutilização do dado afeta a implementação de ações efetivas, principalmente em nível municipal, que possibilitem a prevenção de doenças e agravos relacionados à alimentação e nutrição.^19^

É comum a utilização de dados dos relatórios públicos do Sisvan Web para avaliar o estado nutricional e o consumo alimentar de indivíduos nas diferentes fases da vida;^4,20^ porém, ainda é incipiente a utilização dos dados individualizados que permitam conhecer o perfil alimentar e nutricional dos usuários acompanhados na APS e a interação de seus aspectos pessoais e do ambiente.^21^ A capacidade de vincular diferentes fontes de dados (por exemplo, cuidados primários e dados da atenção especializada) é de suma importância para melhorar a troca de informações sobre saúde, a tomada de decisões, o desenvolvimento de políticas e de produtos e serviços.^22^ Esse relacionamento de banco de dados, também conhecido como *linkage*, ainda não é amplamente difundido nos ambientes da vigilância em saúde; e, quando utilizado, parte da utilização de dados secundários dos sistemas de informação, mas, ainda assim, possibilita vantagens para os serviços de saúde.^13^

Neste estudo, o *linkage* determinístico utilizado entre o banco de antropometria e o banco de consumo alimentar consistiu na presença de chaves unificadoras comuns em bancos de dados distintos, a fim de se construir uma base única com o histórico antropométrico e nutricional.^23^

Essa estratégia de análise técnica, e de consistência entre múltiplas medidas em bancos de coortes, é de suma importância para a garantia de sua qualidade, ainda mais quando operacionalizados bancos de grandes conjuntos de dados.^13^ Também permite a identificação de erros e inconsistências no preenchimento e na digitação, uma limitação comum entre estudos que envolvem esses tipos de dados.^24,25^

Para este estudo, o primeiro banco de dados (antropometria) foi utilizado como referência e o segundo (consumo alimentar) como fonte de novas informações; assim, a identificação de chaves únicas entre as bases foi fundamental para o rastreamento de informações referentes ao mesmo indivíduo. O pareamento das informações permite análises específicas para avaliar associações, colaborando para um olhar ampliado das informações, evitando o uso delas de forma isolada. Com base nas variáveis disponíveis para cada pessoa no banco de dados gerado, torna-se possível a inclusão de covariáveis importantes, que até então não estavam presentes no conjunto de dados original. Elas podem ser obtidas de diferentes áreas, como a social, a ambiental e a de saúde.^25^

Um estudo de coorte retrospectivo avaliou a influência de fatores perinatais no desenvolvimento da obesidade em crianças e adolescentes no sul do Brasil com dados de antropometria registrados no Sisvan Web e dados perinatais registrados no Sistema de Informação de Nascidos Vivos (SINASC). A ligação entre os dois sistemas permitiu a identificação desses fatores de risco para o desenvolvimento da obesidade infantil.^5^

Outro estudo, realizado com dados individualizados de adolescentes de 10 a 19 anos participantes do estudo VigiNUTRI Brasil, no ano de 2018, investigou a associação de fatores contextuais socioeconômicos do município de residência, comportamentos e consumo alimentar com a prevalência de obesidade. Nesse estudo, os fatores contextuais dos municípios foram extraídos de sítios eletrônicos e agregados ao banco do Sisvan, o que aprimorou o escopo de análises possíveis. Assim, foram observadas maiores prevalências de obesidade em adolescentes residentes em municípios com maior renda *per capita* e que consumiram hambúrguer e carnes processadas (marcadores do consumo de alimentos ultraprocessados) no dia anterior ao registro.^21^

Apesar de as metodologias para o *linkage* dos estudos supracitados^5,21^ serem distintas, é possível compreender a necessidade de investimento e incentivo para o uso de bancos de dados de sistemas de informação públicos, pois, além do baixo custo, eles fornecem uma gama de informações do nascimento ao envelhecimento, que podem ser utilizadas para a avaliação de território, tomada de decisões, planejamento e desenvolvimento de ações. Pontua-se que são imprescindíveis mais esforços e sensibilização para a inserção dos dados no sistema, ampliando-se sua cobertura total para todas as fases da vida.^24^

Ademais, o investimento em tecnologia é essencial para a garantia de equipamentos de informática e conexões de internet adequadas, principalmente em municípios menores. Por parte do Ministério da Saúde, é fundamental a manutenção e o aprimoramento do sistema, evitando sua instabilidade, pois a qualidade dos bancos de dados originais será determinante para a qualidade dos bancos vinculados.^13^

O investimento em capacitações e treinamentos para as equipes da APS deve ser realizado, a fim de sensibilizar as equipes para a importância do registro periódico do sistema não só para o módulo de antropometria, mas também para o módulo de consumo alimentar, que ainda possui um volume de registros muito inferior comparado ao de antropometria. 

Entre as limitações identificadas, aponta-se a ausência de estudos prévios, o que levou à decisão pelos pesquisadores da adoção do parâmetro de 30 dias de diferença entre os registros dos adolescentes acompanhados, para a análise técnica e os ajustes dos bancos. Sugere-se que essa seja testada futuramente. Outra limitação é a inviabilidade da inspeção de todos os registros, devido ao grande número e à perda de muitos dados por baixa cobertura, digitação incorreta e descontinuidade do envio dos dados. Embora haja limitações, este estudo se destaca por ser o primeiro a apresentar a descrição de todas as etapas de tratamento de dados individualizados do Sisvan, e espera-se que esse seja o ponto de partida para a análise dos dados individualizados nas demais fases da vida. 

Ao se realizar a junção dos bancos de dados de antropometria e o consumo alimentar de adolescentes acompanhados pelo Sisvan Web entre 2008 e 2018, foi possível vincular e agregar dados individuais, gerando um banco com informações com o perfil alimentar e nutricional para o mesmo indivíduo. 

O Sisvan é reconhecido como instrumento fundamental para a gestão da PNAN no país. O uso de suas informações para a ação é essencial para ampliar o atendimento à população, com foco em fornecer subsídios para políticas de saúde resolutivas que apoiem a tomada de decisões de profissionais e gestores. A descrição aqui delineada permite a realização de atividades de pesquisa, estudos descritivos e analíticos que poderão trazer um panorama do perfil epidemiológico da população acompanhada na APS de forma efetiva.
